# A Novel Standardized *Cannabis sativa* L. Extract and Its Constituent Cannabidiol Inhibit Human Polymorphonuclear Leukocyte Functions

**DOI:** 10.3390/ijms20081833

**Published:** 2019-04-13

**Authors:** Alex Mabou Tagne, Franca Marino, Massimiliano Legnaro, Alessandra Luini, Barbara Pacchetti, Marco Cosentino

**Affiliations:** 1 Center for Research in Medical Pharmacology, University of Insubria, 21100 Varese (I), Italy; amaboutagne@uninsubria.it (A.M.T.); franca.marino@uninsubria.it (F.M.); massimiliano.legnaro@uninsubria.it (M.L.); alessandra.luini@uninsubria.it (A.L.); 2 Linnea SA, CH-6595 Riazzino, TI (CH), Switzerland; bpacchetti@linnea.ch

**Keywords:** cannabidiol, cannabis, neutrophils, inflammation, TNF-α, reactive oxygen species, cell migration

## Abstract

Cannabis and cannabinoids offer significant therapeutic benefits for a wide scope of pathological conditions. Among them, the clinical issues rooted in inflammation stand out, nonetheless, the underlying mechanisms are not yet plainly understood. Circumstantial evidence points to polymorphonuclear leukocytes (PMN) as targets for the anti-inflammatory effects of cannabis. Therefore, we conducted this study to assess the effects of CM5, a novel *Cannabis sativa* L. extract standardized in 5% cannabidiol (CBD), on human PMN functions, including cell migration, oxidative metabolism and production of tumour necrosis factor (TNF)-α. We then sought to investigate whether such effects could be ascribed to its content in CBD. Cell migration was assessed by the Boyden chamber assay, oxidative metabolism by means of spectrofluorimetric measurement of reactive oxygen species (ROS) production, and TNF-α was measured by real time polymerase chain reaction (PCR) and enzyme-linked immunosorbent assay (ELISA). Results show that both CM5 and CBD inhibit PMN migration, ROS and TNF-α production, indicating that CBD may be the main item responsible for the effects of CM5. CM5 is however more potent than CBD on cell migration and TNF-α production, and less effective on ROS production, suggesting that beyond CBD, other components of the cannabis plant may contribute to the biological effects of the extract. As a whole, such results support the use of cannabis standardized extract and CBD to stem inflammation; however, they also warrant in-depth investigation of the underlying cellular and molecular mechanisms to better exploit their therapeutic potential.

## 1. Introduction

**Figure 1:**
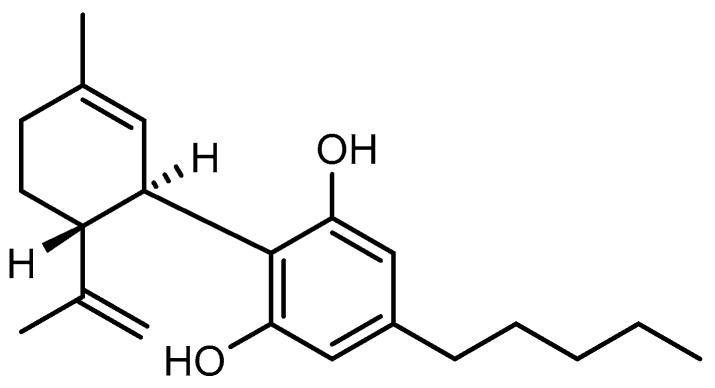
Chemical structure of cannabidiol.

Cannabis (*Cannabis sativa* L., fam. Cannabaceae) is accredited with several medicinal properties [1], and conclusive evidence gives support to its therapeutic benefits in the treatment of chronic pain, multiple sclerosis, as well as chemotherapy-induced nausea and vomiting [2]. Currently, medical cannabis is available in many countries, however, a world of controversy still surrounds cannabis use in clinical practice in consideration of its mind-altering properties and addictive potential related to its ∆^9^-tetrahydrocannabinol (THC) content [3]. As a result, new varieties of cannabis have been developed which are poor in THC and rather rich in non-psychoactive cannabinoids [4]. Cannabidiol (CBD, Figure 1) is the major non-psychoactive cannabinoid and occurs naturally in appreciable amounts in the leaves, and flowers of cannabis plants [5]. CBD is devoid of any drug abuse liability [6] and carries no meaningful side effects across a wide dose range in humans (up to 6000 mg/day p.o.) [7,8,9]. CBD has recently received Food and Drug Administration (FDA) approval for seizures associated with Lennox-Gastaut syndrome or Dravet syndrome [10], furthermore available evidence suggests that CBD might be beneficial in a number of diseases, including: Alzheimer’s disease, Parkinson’s disease, multiple sclerosis, epilepsy, Huntington’s disease, hypoxia-ischemia injury, pain, anxiety, depression, cancer, nausea, inflammatory diseases, infections, rheumatoid arthritis, inflammatory bowel and Crohn’s diseases, cardiovascular disease and diabetic complications [11]. Preliminary studies suggest that most of the therapeutic effects of cannabis and CBD may be linked to their ability to affect immunity and inflammation [12], nonetheless the underlying mechanisms are not yet plainly understood.

Polymorphonuclear leukocytes (PMN) play a critical role in the inflammatory process as the first-line of defence against invading microorganisms [13]. PMN are sensitive to agents such as bacterial by-products as well as to cytokines and chemokines [14]. Thereafter, migrating into the site of inflammation, they produce and release proinflammatory and antimicrobial mediators, including cytokines and chemokines, as well as oxidative metabolites such as reactive oxygen species (ROS). Rapid resolution of inflammation usually leads to tissue repair, however chronicization of the process results in tissue damage, which is considered pivotal in a wide range of inflammatory diseases [15]. PMN are therefore attractive targets for the development of novel anti-inflammatory therapeutics in many different clinical settings [16,17].

Circumstantial evidence suggests that inhibition of PMN functions could be a potential mechanism underlying the anti-inflammatory effects of CBD. Indeed, in mice, it has been reported that CBD suppresses PMN polarization towards the proinflammatory N1 phenotype [18], attenuates myeloperoxidase activity [19], and inhibits respiratory burst [20]. Evidence in human PMN includes so far inhibition of oxidative metabolism [20], adhesion to the activated endothelium [21] as well as cell migration [20,22]. On the other hand, however, there is no evidence regarding the activity of cannabis on PMN. Therefore, the present study was conducted to examine the effects of CM5, a *Cannabis sativa* L. extract standardized in 5% CBD and with a low content of THC (<0.2%), on human PMN functions, including cell migration, oxidative metabolism and production of tumour necrosis factor (TNF)-α. We then systematically compared CM5 with pure CBD, in order to determine whether the effects of cannabis on PMN could be ascribed to its content in CBD.

## 2. Results

### 2.1. Effect of CM5 and Cannabidiol (CBD) on Cell Viability

**Figure 2:**
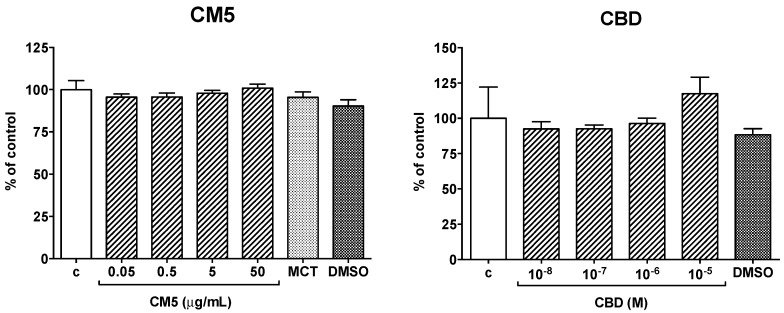
Effect of CM5 and cannabidiol (CBD) and of their respective mediums, medium chain triglycerides (MCT) and dimethylsulfoxide (DMSO), on polymorphonuclear leukocytes (PMN) viability. PMN were cultured for 21 h alone or in the presence of the test substances at the indicated concentrations. MCT and DMSO were added at concentrations equivalent to those contained in CM5 50 μg/mL and in CBD 10 μM, respectively. Each column is the mean ± SEM of 5 separate preparations in duplicate. c = control.

Treatment of human PMN with CM5 0.05–50 μg/mL or CBD 0.01–10 μM, as well as with their respective mediums, medium chain triglycerides (MCT) and dimethylsulfoxide (DMSO), carried no meaningful effect on cell viability (Figure 2).

### 2.2. Effect of CM5 and CBD on Cell Migration

**Figure 3:**
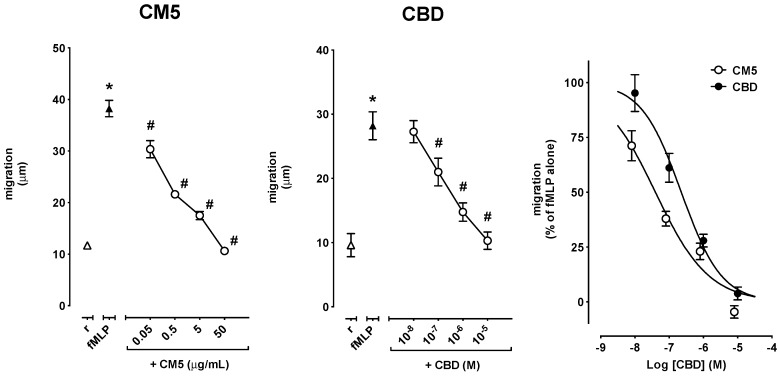
Effect of CM5 and CBD on fMLP-induced PMN migration shown as absolute values (left and centre panels) and as the percent of inhibition of migration of fMLP–stimulated PMN (right panel). Values are mean ± standard error of the mean (SEM) of 5–11 separate observations. * *p* < 0.0001 vs. resting and # *p* < 0.001 vs. fMLP alone.

**Figure 4:**
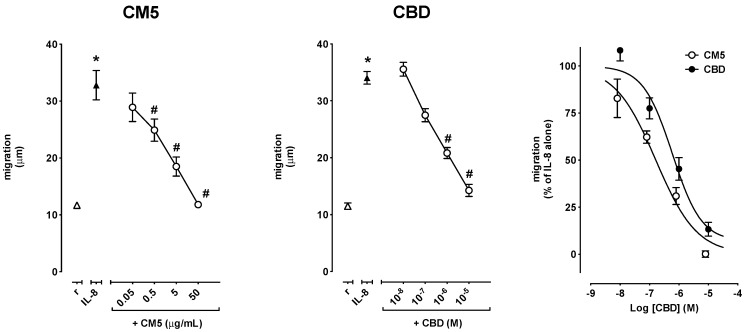
Effect of CM5 and CBD on IL-8-induced PMN migration shown as absolute values (left and centre panels) and as the percent of inhibition of migration of fMLP–stimulated PMN (right panel). Values are mean ± SD of at least 5–13 experiments. * *p* < 0.001 vs. resting and # *p* < 0.001 vs. IL-8 alone.

PMN migration was increased by fMLP as well as by IL-8. In particular, 0.1 μM fMLP increased migration from 11.6 ± 0.5 μm in resting cells up to 37.0 ± 1.4 μm (*n* = 8–11, *p* < 0.0001) in experiments devised to test the effect of CM5, and from 10.3 ± 1.1 μm in resting cells to 30.2 ± 1.8 μm (*n* = 5–8, *p* < 0.001) in experiments devised to test the effect of CBD (Figure 3), while 10 ng/mL IL-8 increased migration from 11.7 ± 0.3 μm in resting cells up to 32.8 ± 2.6 μm (*n* = 5, *p* < 0.001) in experiments devised to test the effect of CM5, and from 11.9 ± 0.4 μm in resting cells to 34.8 ± 1.0 μm (*n* = 9–12, *p* < 0.0001) in experiments devised to test the effect of CBD (Figure 4). CM5 and CBD concentration-dependently reverted the effect of fMLP and IL-8 down to control values with CM5 50 μg/mL and CBD 10 μM (Figure 3 and Figure 4, left and centre panels). Comparison of the Log concentration-response curves for CBD and CM5 (expressed as molar concentrations of CBD) showed, however, that CM5 was significantly more potent than CBD on fMLP-induced migration of PMN (Figure 3, right panel), as also indicated by the lower IC_50_ (Table 1). In comparison to the Log concentration-response curve of CBD, the curve of CM5 was shifted to the left also for IL-8-induced cell migration (Figure 4, right panel), however, the difference between the respective IC_50_s did not reach the statistical significance (Table 1). In resting conditions, neither CM5 nor CBD affected PMN migration at any of the concentrations tested (data not shown).

### 2.3. Effects of CM5 and CBD on Reactive Oxygen Species (ROS) Production

**Figure 5:**
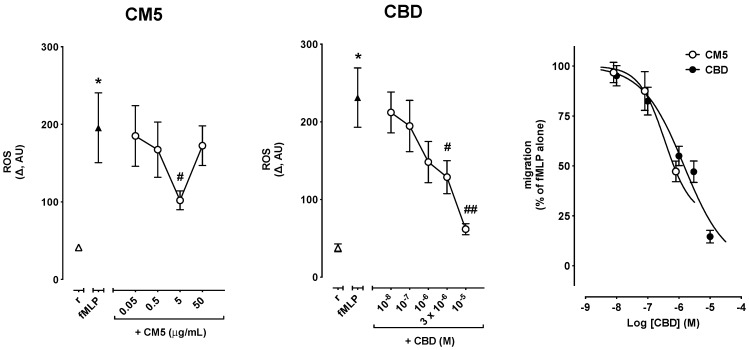
Effect of CM5 and CBD on fMLP-induced reactive oxygen species (ROS) production in PMN. ROS changes were calculated over 30 min as the difference (Δ) between resting levels and peak levels induced by fMLP. * *p* < 0.001 vs. resting, # *p* < 0.05 and ## *p* < 0.001 vs. fMLP alone.

ROS production was increased by 0.1 μM fMLP from 41.3 ± 3.8 AU in resting cells to 195.6 ± 45.1 AU (*n* = 8, *p* < 0.01) in experiments devised to test the effect of CM5, and from 37.8 ± 5.1 AU in resting cells to 231.2 ± 38.2 AU (*n* = 9, *p* < 0.001) in experiments devised to test the effect of CBD (Figure 5). Coincubation with CM5 up to 50 μg/mL or with CBD up to 10 μM did not affect fMLP-induced ROS production (data not shown). By contrast, preincubation for 1 h resulted in a concentration-dependent attenuation of fMLP-induced ROS production with both CM5 and CBD (Figure 5, left and centre panels). CM5 however reached the maximum effect at 5 μg/mL, while CM5 50 μg/mL failed to modify the effect of fMLP (Figure 5, left panel). On the other hand, CBD inhibited fMLP-induced ROS production down to control levels with CBD 10 μM (Figure 5, centre panel). Comparison of the concentration-response curves of CBD and CM5 (expressed as molar concentrations of CBD) showed however that CM5 was slightly more potent than CBD (Table 1). However, the maximum inhibitory effect observed with CM5 5 μg/mL was still 37.8% of the effect of fMLP alone, while the maximum inhibitory effect of CBD 10^−5^ M completely suppressed the response to fMLP (Figure 5, right panel).

Remarkably, in resting cells, CM5 did not affect ROS levels up to 5 μg/mL, however 50 μg/mL resulted in a huge increase of ROS production (from 59.9 ± 4.0 AU in resting to 252.6 ± 32.9 AU, *n* = 8, *p* < 0.001). Likewise, CBD did not significantly affect resting ROS levels, although CBD 10^−5^ M resulted in increased ROS production which however did not reach the statistical significance (71.4 ± 10.4 AU in resting to 130.2 ± 28.4 AU, *n* = 9, *p* = 0.072).

### 2.4. Effects of CM5 and CBD on TNF-α Production

**Figure 6:**
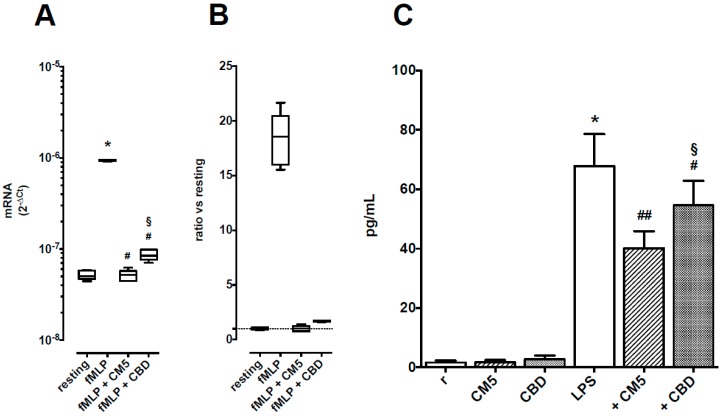
Effects of CM5 50 μg/mL and CBD 10 μM on TNF-α production in PMN. Panel A: TNF-α mRNA levels, shown as medians with 25th–75th percentiles (boxes) and min–max values (whiskers) of 5 separate observations. * *p* < 0.001 vs. resting, # *p* < 0.001 vs. fMLP alone and ^§^ *p* < 0.05 vs. CM5. Panel B: TNF-α mRNA levels, shown as ratio vs. resting cells. Panel C: TNF-α protein levels in supernatants from cultured PMN. Each column is the mean ± SEM of 10 separate observations. * *p* < 0.001 vs. resting, # *p* < 0.05 and ## *p* < 0.01 vs. LPS alone and § *p* < 0.05 vs. CM5.

Levels of TNF-α mRNA were upregulated in stimulated PMN by nearly 20-fold (Figure 6, panels A and B) and TNF-α protein levels in supernatants from resting cells were 1.7 ± 2.1 pg/mL and raised up to 67.6 ± 34.3 pg/mL after PMN activation (Figure 6, panel C). CM5 50 μg/mL and CBD 10 μM reduced TNF-α mRNA levels down to control values, although the inhibitory effect of CM5 was higher than that of CBD (*p* < 0.05 vs. CBD, *n* = 5). CM5 50 μg/mL and CBD 10 μM had no effect on TNF-α protein levels in supernatants from resting cells, but reduced TNF-α production in activated PMN, again with CM5 being more effective than CBD (*p* < 0.05, *n* = 10).

## 3. Discussion

The present study provides evidence that CM5, a standardized cannabis extract in CBD 5% and with THC <0.2%, extensively affects human PMN functions. Specifically, at non-cytotoxic concentrations, CM5 was found to effectively inhibit stimulated migration, oxidative metabolism and production of the proinflammatory cytokine TNF-α. Comparison with the effects of pure CBD suggests that most of the activity of CM5 could be explained by its CBD content. However, CM5 at equimolar concentration of CBD was more potent and effective than CBD alone on PMN migration, as well as on TNF-α production. Moreover, CBD alone was more effective than CM5 on PMN oxidative metabolism.

To our best knowledge, this is the first study reporting the inhibitory effects of a cannabis extract rich in CBD and with a low level of THC on human PMN functions. With respect to CBD, however, our findings support previous research showing the ability of CBD to inhibit the oxidative metabolism [20] and to attenuate PMN migration [22], and extend for the first time the observations to TNF-α production. Importantly, the inhibitory effects of CBD occurred at concentrations achieved with doses used clinically for the treatment of drug-resistant seizures in Lennox–Gastaut syndrome or Dravet syndrome (up to 20 mg per kg of body weight per day) [23], suggesting that our findings can be easily translated into clinics.

CM5 is a complex mixture containing 5% of CBD and 95% of cannabis vegetal complex matrix (including compounds such as other cannabinoids, flavonoids, fatty acids). Our study revealed that CBD alone reproduces the inhibitory activity of CM5 on human PMN functions, suggesting that the effects of CM5 on PMN could be ascribed chiefly to its CBD content. However, we observed subtle differences in terms of potency and efficacy between CM5 and CBD, indicating that beyond CBD, other components occurring in CM5 may contribute to its inhibitory effects on PMN. Thus, the higher activity of CM5 on PMN migration and TNF-α production may result from synergistic and/or additive interactions between its various components. On the other hand, the lower activity of CM5 on oxidative burst may stem from antagonistic interactions. Further research is, therefore, needed for the identification, isolation as well as pharmacological characterization of components present in cannabis and effectively contributing to its overall activity on PMN.

Deciphering the mechanism underlying the inhibitory effects of CM5 and CBD on PMN functions was beyond the scope of our study. However, clues from prior research suggest that CBD may exert its effects on PMN possibly through CB_2_, at least in rodent models [24], but also by acting on other molecular targets distinct from CB_1_ and CB_2_ [22]. Future mechanistic studies to determine the molecular basis of the inhibitory effects of CBD on PMN should focus on CBD targets that are expressed on human PMN such as CB_2_ [25], GPR55 [26], PPARγ [27] and TRPV1 [28].

PMN are recruited to sites of inflammation where they eradicate pathogens through various effector mechanisms including degranulation, phagocytosis, generation of ROS as well as neutrophil extracellular traps (NETs) formation [29,30,31]. However, excessive PMN responses may contribute to ongoing inflammation and tissue damage in a number of diseases and ailments [32,33,34,35]. Compelling evidence supports the use of cannabis and CBD in a plethora of pathological conditions, some of which stem from the exacerbation of the inflammatory response of PMN and include pain [36], multiple sclerosis [37], diabetic complications [38], inflammatory bowel disease [39], cardiovascular diseases [40], hypoxia-ischemia injury [41], rheumatoid arthritis [35], cancer [42], and Crohn’s disease [43]. Collectively, our findings point to PMN as potential targets for the therapeutic actions of cannabis and CBD.

Since PMN activation results in a much more complex pattern of functional changes that, in addition to activation of migration, respiratory burst and cytokine production, also include phagocytosis, production and release of proteolytic enzymes and NETs as well as interaction with other cells, a great deal of knowledge is still required before a firm conclusion can be drawn about the inhibitory effects of CM5 and CBD on PMN. Moreover, as the effects on TNF-α were not so intense as those on migration and oxidative metabolism, any difference in receptor and/or signal transduction pathways involved might deserve consideration. In any case, available evidence is encouraging and now strongly warrants careful in-depth characterization in order to provide a rational basis for better exploiting the potential health benefits of cannabis or its derivative in broad-impact pathological conditions including pain, diabetic complications, cancer and cardiovascular diseases.

## 4. Materials and Methods

### 4.1. Test Substances

*Cannabis sativa* L. extract oil containing 5% CBD in medium chain triglycerides (MCT, dark green viscous liquid, batch n° 74717009) and pure CBD (white/off-white or slightly yellow powder, batch n° 74717009) were kindly provided by LINNEA SA (https://www.linnea.ch/). Certificates of analysis of both substances are provided as supporting information (Figure S1). Stock solutions were prepared in dimethylsulfoxide (DMSO, Sigma-Aldrich, St. Louis, MI, USA, code: 276855) at concentrations of CM5 50 mg/mL and CBD 10^−2^ M, covered tightly with tinfoil, and stored for up to 1 month at 4 °C and −20 °C, respectively. Stock solutions were diluted in either Hanks’ Balanced Salt Solution (HBSS: NaCl 0.1448 M, KCl 5 × 10^−3^ M, MgSO_4_ 2.04 × 10^−3^ M, CaCl_2_ 1.32 × 10^−3^ M, glucose 10^−2^ M) modified with 10^−2^ M HEPES (HBSS/HEPES) or RPMI medium (Euroclone, code: ECM0495L) as required by the experimental procedures.

### 4.2. Isolation of Human Polymorphonuclear Leukocytes (PMN)

Human PMN were obtained from buffy coats of blood donations through the courtesy of the local blood bank (Ospedale di Circolo, Fondazione Macchi, Varese, Italy). In brief, PMN were isolated by Dextran sedimentation followed by Ficoll-Paque Plus (GE Healthcare, Milan, Italy, code: GEH17144003) density-gradient centrifugation, as described previously [44]. Contaminating erythrocytes and platelets were eliminated by 5-min hypotonic lysis in distilled water with added NH_4_Cl 8.3 g/L, KHCO_3_ 1.0 g/L, and ethylenediamine tetraacetic acid 37 mg/L. Cells were then washed twice in NaCl 0.15 M. Experiments were performed only when the purity and viability of isolated PMN, as assessed by light microscopy, were over 95%.

### 4.3. Cytotoxicity Assays

Cytotoxicity of test substances was assessed on PMN by means of the MTT [3-(4,5-dimethyl-2-thiazolyl)-2,5-diphenyl-2H tetrazolium bromide] reduction method [45]. In short, freshly isolated PMN were resuspended at 1 × 10^6^ cells/mL in RPMI 1640 medium supplemented with 10% foetal bovine serum (FBS, Euroclone, code: ECS0180 L) and 1% penicillin/streptomycin (Euroclone, code: ECB 3001D). Cells were then seeded in duplicate in a 96-well round bottom plate (250 μL of suspension per well) and cultured for 24 h alone or in the presence of a test substance at 37 °C in 5% CO_2_ atmosphere. The absorbance (optical density, OD, in arbitrary units) was measured using a microplate spectrophotometer with a 570 nm test wavelength and a 690 nm reference wavelength. Results were expressed as mean OD value of duplicates.

### 4.4. Cell Migration Assay

PMN migration was investigated by the Boyden chamber assay modified as previously described [46]. Briefly, after instrument assembly, PMN at 1 × 10^6^ cells/mL in RPMI 1640 medium were placed in the upper chamber alone or together with the test substance, while the lower chamber contained medium alone (spontaneous migration) or added with 10 ng/mL interleukin (IL)-8 (Sigma–Aldrich, code: I1645) or 0.1 μM N-formyl-Met-Leu-Phe (fMLP, Sigma–Aldrich, code: F3506) (stimulated migration). Chambers were separated by a 3 μm pore-sized filter. After a 90-min incubation at 37 °C, the filter was harvested, dehydrated, fixed, and finally stained with haematoxylin. PMN migration was then quantified by light microscopy measuring the distance (in μm) from the surface of the filter to the leading front of cells.

### 4.5. Reactive Oxygen Species (ROS) Production Assay

Intracellular ROS production was assayed by use of the redox-sensitive dye C-DCFH-DA (Molecular probes, Eugene, OR, USA, code: C2938) as previously described [47]. Briefly, fluorescence was measured by means of a spectrofluorimeter (PerkinElmer LS-50B, PerkinElmer Instruments, Bridgeport, CT, USA), set at 488 nm excitation wavelength and 525 nm fluorescence emission. The effects of CM5 and CBD were tested on resting cells and on cells stimulated with 0.1 μM fMLP. In each experiment, C-DCFH-DA-stained PMN resuspended at 1 × 10^6^ cells/mL in HBSS/HEPES were placed in the spectrofluorimeter and the test substances were added after a 60-s resting period. ROS changes, expressed as fluorescence intensity in arbitrary units (AU), were calculated as the difference (Δ) between resting levels and peak levels induced by the treatment over a 30-min period.

### 4.6. Measurement of TNF-α Production

Freshly isolated PMN were resuspended at 1 × 10^7^ cells/mL in RPMI 1640 medium supplemented with 10% FBS and 1% penicillin/streptomycin and placed in sterile 5-mL test tubes. Cells were then stimulated with lipopolysaccharide (LPS) from *E. coli* serotype O127:B8 (1 μg/mL; L3137, Sigma–Aldrich) or 0.1 μM fMLP and cultured for up to 21 h alone or in the presence of the test substances at 37 °C under a 5% CO_2_ atmosphere. After incubation, cells were centrifuged (1500× *g*, 10 min, 20 °C) and pellets and supernatants were harvested and stored at −80 °C until further analyses were performed.

Cell pellets were used for quantification of mRNA levels of TNF-α using real-time reverse transcription polymerase chain reaction (RT-PCR). At least 5 × 10^4^ PMN were resuspended in PerfectPure RNA lysis buffer (5Prime™ GmbH, Hamburg, Germany). Next, total RNA was extracted by Aurum™ total RNA mini kit (BIO-RAD, code: 732-6470) and the amount of extracted RNA was estimated by spectrophotometry at λ = 260 nm. Total mRNA was then reverse-transcribed using a random primer and high-capacity cDNA RT kit (Life Technologies Corporation, code: 4368813), and the resulting amount of cDNA was estimated by spectrophotometry at λ = 260 nm. Real-Time PCR reactions were then started with 1 μM cDNA. Amplification of cDNA was performed by means of SsoAdvanced™ Universal Probes Supermix (BIO-RAD, code: 1725282) for the analysis of mRNA levels of TNF-α under conditions depicted in Table S1 provided as supporting information. cDNA was assayed on StepOne^®^ System (Applied Biosystems). Linearity of real-time PCR assays were tested by constructing standard curves by use of serial 10-fold dilutions of a standard calibrator cDNA for TNF-α gene. Regression coefficients (r^2^) were always >0.999 (data not shown). The level of TNF-α mRNA in a given sample was represented as 2^−∆*C*t^ where ∆*C*t = [*C*t (sample) − *C*t (housekeeping gene)]. Relative expression was determined by normalization to expression of *RPS18*, which is the gene for 18S cDNA. Data analysis was performed by StepOne software™ 2.2.2 (Applied Biosystems).

TNF-α protein levels were measured in culture supernatants using commercial enzyme-linked immunosorbent assay (ELISA) kits (Invitrogen, code: KHC0081) according to the protocol supplied by the manufacturer.

### 4.7. Statistical Analysis

Data are shown as mean ± standard error of the mean (SEM), unless otherwise indicated, with *n* showing the number of replicates. Differences between groups were assessed by Student’s *t*-test using MS Excel (Microsoft, 2016). Concentration-response relationships were analysed by non-linear regression using GraphPad Prism version 6.00 for Windows (GraphPad Software, La Jolla, CA, USA, www.graphpad.com). To this end, CM5 concentrations were expressed as CBD content in μM (CM5 stock solution 50 mg/mL ≈ CBD 10^−2^ μM). The mean values of IC_50_ (i.e., the concentration which elicits 50% of the maximal inhibition) were finally calculated together with their 95% confidence intervals (CI). *p* < 0.05 was considered statistically significant.

## Figures and Tables

**Table 1:** IC_50_ values of the Log concentration–response curves of CM5 and CBD on PMN functions. Data are expressed as log (M) of CBD.

PMN Functions	CM5 (Mean ± SEM)	CBD (Mean ± SEM)	*p*-Value
fMLP-induced migration	−7.401 ± 0.111	−6.645 ± 0.106	0.0008
IL-8-induced migration	−6.786 ± 0.124	−6.225 ± 0.219	0.0648
fMLP-induced ROS production	−6.449 ± 0.148	−5.832 ± 0.089	0.0023

## Supplementary Materials

Supplementary materials can be found at https://www.mdpi.com/1422-0067/20/8/1833/s1.
