# Supplementary material for: A Novel Standardized Cannabis sativa L. Extract and Its Constituent Cannabidiol Inhibit Human Polymorphonuclear Leukocyte Functions
Source: Int J Mol Sci. 2019 Apr 13;20(8):1833. doi: 10.3390/ijms20081833 (PMC6515348; doi:10.3390/ijms20081833)
Supplement: Supplementary file 1 [file ijms-20-01833-s001.zip › Figure S1.pdf]

Linnea

Code	Sale specification: SVPF	Version n. 2
**CM5**	**Cannabis sativa extract 5**	**Effective date: 11.01.2018**<br>**Discard any previous version**

**Commercial name:**	Cannabis sativa extract 5
**Scientific name:**	*Cannabis sativa L.* extract
**CAS Number:**	89958-21-4
**Chemical name (cannabidiol):**	2-[(1R, 6R)-6-isopropenyl-3-methyl-2-cyclohexen-1-yl]-5-pentyl-1,3-benzenediol
**Molecular formula (cannabidiol):**	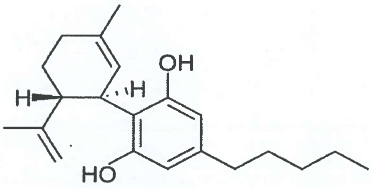 $C_{21}H_{30}O_2$
**Molecular weight (cannabidiol):**	314.5 g/mol
**Description:**	Dark green viscous liquid
**Storage and packaging:**	Preserve in tight container, protected from light, and humidity. Do not store above 25°C.<br>HDPE wide neck drum with screw cap.

**Test**	**Limit**	**Method**
Appearance	Dark green viscous liquid	In-house (visual)
Identification (HPLC)	Complies	In-house
Cannabinoids:		
Sum of CBD and CBDA, expressed as cannabidiol (on as-is basis)	5.0 ± 0.5 %	In-house (HPLC)
Sum of THC and THCA (on as-is basis)	≤ 0.2 %	
Residual solvents:		In-house (GC)
Ethanol	Not more than 5000 ppm	
Water content (KF)	≤ 1.0 %	Ph Eur (2.5.12) current ed.
Microbial contamination:		
Total aerobic microbial count	Not more than 10^3 cfu/g	Ph Eur (2.6.12) current ed.
Total combined yeast/mould count	Not more than 10^2 cfu/g	Ph Eur (2.6.12) current ed.
Bile-tolerant Gram-negative bacteria	Not more than 10^2 cfu/g	Ph Eur (2.6.13) current ed.
Escherichia coli	Absent/10 g	Ph Eur (2.6.13) current ed.
Salmonella	Absent/25 g	Ph Eur (2.6.31) current ed.

Remarks: medium chain triglycerides are used as carrier

Linnea

Code	Sale specification: SVPF	Version n. 2
**CM5**	**Cannabis sativa extract 5**	**Effective date: 11.01.2018**<br>**Discard any previous version**

Prepared by:
S. Vincenti/ Regulatory Affairs 11/01/2018

Approved by:
G.Tossi / Technical Director 11/01/18

Customer's Approval

We accept all the above specification for the product :

Signed by: ....

Function: ....

Company: ....

Date: ....

Code	Sale specification: SVPF	Version N 1
**CBD**	**Cannabidiol**	**Effective date: 10.08.2018**<br>**Discard any previous version**

**Trade name:**	Cannabidiol
**Scientific name:**	Cannabidiol
**CAS number**	13956-29-1
**Chemical name:**	2-[(1*R*,6*R*)-6-isopropenyl-3-methylcyclohex-2-en-1-yl]-5-pentylbenzene-1,3-diol
**Molecular formula :**	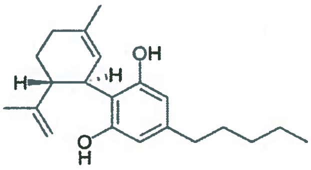
**Molecular weight:**	314.5 g/mol
**Description:**	White/off-white or slightly yellow powder
**Storage and packaging:**	Preserve in tight container, protected from light and humidity. Do not store above 25°C.<br>Double polyethylene, into plastic drums.

Test	Limit	Method
Appearance	White/off-white or slightly yellow powder	In-house (visual)
Identification (HPLC)	Complies	In-house
Identification (IR)	Complies	Ph. Eur. (2.2.24) current ed.
Assay (dried substance)	Between 98.0 % and 102.0 %	In-house (HPLC)
Related substances:		In-house (HPLC)
Total THC (sum of Δ8-THC and Δ9-THC)	Not more than 0.10%	
CBDV	Not more than 0.5%	
CBD-C4	Not more than 0.5%	
Other related substances (each)	Not more than 0.10%	
Related substances (total)	Not more than 1.0%	
Melting point	Between 65°C and 69°C	Ph. Eur. (2.2.14) current ed.
Water content	Not more than 1.0%	Ph. Eur. (2.5.12) current ed.
Sulphated Ash	Not more than 0.1%	Ph. Eur. (2.4.14) current ed.
Specific Optical Rotation<br>*(on dried substance)*	Between -135.0 and -125.0	In-house
Residual solvents		In-house (GC)
Ethanol	Not more than 5000 ppm	
Hexane	Not more than 290 ppm	
Microbiology		
Total aerobic microbial count	Not more than 10^3 CFU/g	Ph. Eur. (2.6.12) current ed.
Total combined yeast/mould count	Not more than 10^2 CFU/g	Ph. Eur. (2.6.12) current ed.
Bile-tolerant Gram-negative bacteria	Not more than 10^2 CFU/g	Ph. Eur. (2.6.13) current ed.
Escherichia coli	Absent/10g	Ph. Eur. (2.6.13) current ed.
Salmonella	Absent/25g	Ph. Eur. (2.6.31) current ed.

Code	Sale specification: SVPF	Version N 1
**CBD**	**Cannabidiol**	**Effective date: 10.08.2018**<br>**Discard any previous version**

Remarks:

Prepared by:
D. Maradei / QA Manager 08.08.2018

Approved by:
G.Tossi / Technical Director 080818

Customer's Approval

We accept all the above specification for the product :

Signed by: ....

Function: ....

Company: ....

Date: ....
