# Supplementary material for: A Novel Standardized Cannabis sativa L. Extract and Its Constituent Cannabidiol Inhibit Human Polymorphonuclear Leukocyte Functions
Source: Int J Mol Sci. 2019 Apr 13;20(8):1833. doi: 10.3390/ijms20081833 (PMC6515348; doi:10.3390/ijms20081833)
Supplement: Supplementary file 1 [file ijms-20-01833-s001.zip › Table S1 .pdf]

Supplementary Table S1. Real-Time PCR conditions.

**Gene Symbol**	**UniGene ID**	**Interrogated sequences** *RefSeq/GenBank mRNA*	**Detected coding transcripts**	**Amplicon context sequence**	**Chromosome location**	**Amplicon length**	**Annealing temperature** (°C)	**Efficiency** (%)
TNFA	Hs.241570	NC_000006.11<br>NG_007462.1<br>NG_012010.1<br>NT_007592.15<br>NT_113891.2<br>NT_167244.1<br>NT_167245.1<br>NT_167246.1<br>NT_167247.1<br>NT_167248.1<br>NT_167249.1	ENST00000328965<br>ENST00000445232<br>ENST00000594551<br>ENST00000443707<br>ENST00000412275<br>ENST00000449264<br>ENST00000577810<br>ENST00000326294<br>ENST00000448781<br>ENST00000420425<br>ENST00000394126<br>ENST00000356271<br>ENST00000394128<br>ENST00000394127<br>ENST00000422942<br>ENST00000501516<br>ENST00000536318<br>ENST00000431269<br>ENST00000376122<br>ENST00000383496<br>ENST00000264203<br>ENST00000375144<br>ENST00000375142<br>ENST00000401084<br>ENST00000439554	GGGGTCTTCCAGCTGGAGAAGGGTGA<br>CCGACTCAGCGCTGAGATCAATCGGC<br>CCGACTATCTCGACTTTGCCGAGTCTG<br>GGCAGGTCTACTTTGGGATCATTGCCCT<br>GTGAGGAGGACGAACATC	6:31545204-31545328	95	60	99
RPS18	Hs.627414	NC_000006.11<br>NT_007592.15<br>NT_113891.2<br>NT_167245.1<br>NT_167247.1<br>NT_167248.1<br>NT_167249.1	ENST00000454021<br>ENST00000486781<br>ENST00000484321<br>ENST00000211372<br>ENST00000477055<br>ENST00000476288<br>ENST00000439602<br>ENST00000474973<br>ENST00000457341<br>ENST00000494232<br>ENST00000434122	GTGGAACGTGTGATCACCATTATGCAGA<br>ATCCACGCCAGTACAAGATCCCAGACTG<br>GTTCTTGAACAGACAGAAGGATGTAAAG<br>GATGGAAAATACA	6:33243742-33243838	67	60	98
